# Ideal Living Skin Equivalents, From Old Technologies and Models to Advanced Ones: The Prospects for an Integrated Approach

**DOI:** 10.1155/2024/9947692

**Published:** 2024-08-16

**Authors:** Andrei Riabinin, Maria Pankratova, Olga Rogovaya, Ekaterina Vorotelyak, Vasiliy Terskikh, Andrey Vasiliev

**Affiliations:** Department of Cell Biology Koltzov Institute of Developmental Biology of the Russian Academy of Sciences, Moscow, Russia

## Abstract

The development of technologies for the generation and transplantation of living skin equivalents (LSEs) is a significant area of translational medicine. Such functional equivalents can be used to model and study the morphogenesis of the skin and its derivatives, to test drugs, and to improve the healing of chronic wounds, burns, and other skin injuries. The evolution of LSEs over the past 50 years has demonstrated the leap in technology and quality and the shift from classical full-thickness LSEs to principled new models, including modification of classical models and skin organoids with skin derived from human-induced pluripotent stem cells (iPSCs) (hiPSCs). Modern methods and approaches make it possible to create LSEs that successfully mimic native skin, including derivatives such as hair follicles (HFs), sebaceous and sweat glands, blood vessels, melanocytes, and nerve cells. New technologies such as 3D and 4D bioprinting, microfluidic systems, and genetic modification enable achievement of new goals, cost reductions, and the scaled-up production of LSEs.

## 1. Introduction

Tissue engineering is a successfully developing, promising area of translational medicine, and its achievements in the foreseeable future can be used both to restore the normal functioning of the original organs and to generate their equivalents, which can be used as models for a wide range of studies. Current cell biology technologies allow using both cells derived from primary cultures isolated from human tissues and cells obtained by directed differentiation of human-induced pluripotent stem cells (iPSCs) (hiPSCs) for these purposes. The human skin, together with its derivatives, is an organ whose morphogenesis is well-studied, with development of technologies for creating its equivalents. The first skin equivalents appeared as early as the second half of the 20th century, but models of living skin equivalents (LSEs) and technologies for their construction have evolved considerably over the past 50 years. LSEs are tissue-engineered structures that simulate the epidermal and dermal components of the human skin. The latest developments in this field make it possible to obtain LSEs that are morphologically and functionally close to their natural counterparts. The main goal of this review is to describe the primary aspects of LSE application, the evolution of main types and generation methods of LSEs, their advantages and disadvantages, and the prospects of combining the advanced technologies for ideal skin graft construction.

## 2. Human Skin and Its Derivatives

### 2.1. Human Skin Components

The human skin is an organ that provides a barrier between the internal environment of the body and the external environment. It consists of epidermis, dermis, hypodermis, and skin derivatives with special functions: hair follicles (HFs), sebaceous and sweat glands, and various receptors [1–3]. When the skin is damaged, the body's main task is the restoration of barrier properties, which is associated primarily with a partial or complete restoration of the structure of the skin, because the structure and functions of this organ are closely related.

### 2.2. Epidermis

The primary function of the epidermis is to provide the main barrier function, preventing chemicals and pathogens in the external environment from entering the dermis and the rest of the body, as well as hindering exposure to mechanical factors and UV [1, 4]. About 95% of the epidermis cell mass is constituted by dense layers of keratinocytes, and the remaining 5% consists of melanocytes, Langerhans cells (immune phagocytic cells), Merkel cells (pain receptors) and free nerve endings (tactile receptors), and cytotoxic CD8^+^ T lymphocytes [1, 4, 5]. Melanocytes provide UV protection [6], while Merkel and Langerhans cells provide immune and receptor functions [1, 4]. These cell types are located in the basal layer of the epidermis. The most important function of the epidermis is protection against dehydration. Mice deprived of a normally functioning epidermis by mutations die shortly after birth [7]. The immune function of the epidermis is related to the ability of epidermal cells to initiate antigen presentation by dendritic cells and Langerhans cells [8]. In addition, keratinocytes synthesize a wide range of cytokines involved in immune responses: interleukins, including proinflammatory ones, growth factors, colony-stimulating factors, and chemokines [9].

The epidermis is separated from the dermis by the basal membrane, which consists mainly of laminins, Type IV collagen, heparan sulfate, and proteoglycans [10]. Epidermal stem cells are anchored directly on the basal lamina and together with their progeny form epidermal proliferative units responsible for epidermal regeneration [11].

### 2.3. Dermis

The dermal layer has regulatory, supportive, and skeletal functions. It includes fibroblasts in the extracellular matrix consisting of elastin fibers and collagen fibers, primarily Collagen Types I and IV, the space between which is filled with glycoproteins and proteoglycans [12]. The matrix is synthesized by fibrocytes and fibroblasts. This layer contains the end sections of exocrine glands, nerves, and blood and lymphatic vessels. In addition to fibroblasts and fibrocytes, the cellular composition consists of mast cells, CD4^+^ helper-induced and regulatory T-lymphocytes, neutrophils, eosinophils, dendritic cells, histiocytes, and various receptor cells [5]. The dermis consists of two layers: an upper papillary layer (papillary dermis), consisting of loose fibrous connective tissue, and a lower reticular layer (reticular dermis) of dense fibrous connective tissue [13]. The dermis provides endocrine and exocrine functions as well as receptor functions [14].

### 2.4. Hypodermis

The hypodermis (subcutaneous fatty tissue) is also called subcutaneous fascia and resembles the dermis structurally, containing a network of collagen and elastin. However, unlike the dermis, the space between the fibers in the hypodermis is filled with adipocytes [15]. Adipocytes within the hypodermis secrete a complex of hormones involved in metabolic regulation. This layer contains blood and lymphatic vessels, nerves, glands, bases of HFs, few collagen and elastin fibers, and Vater–Pacini cells (pressure and vibration receptors). Cellular composition includes adipocytes, fibroblasts, mast cells, and tissue macrophages. The hypodermis performs the functions of heat insulation, shock absorption, and energy reserve (accumulation of fat, glycogen, and water), as well as providing humoral regulation of the body and being involved in the suppression of inflammatory processes [16].

### 2.5. Skin Derivatives

Skin derivatives are isolated derivatives characterized by unique morphology and functions [1]. They either perform secretory functions (sweat, sebaceous, and/or mammary glands, meibomian glands, and ceruminous glands) or produce keratin-containing structures composed of keratinized dead cells: the matrix producing nails and the HF-producing hair [1]. The keratinizing derivatives perform various protective, thermoregulatory, signaling, and sexual functions. The HF simultaneously provides hair growth and development, as well as epithelial cell keratinization products, and is also involved in skin development and self-maintenance [17, 18]. In addition, it participates in sympathetic skin innervation [19].

## 3. Artificial Skin Substitutes and LSEs

Skin equivalents are artificial or cell-generated analogs of one or more layers based on a substrate (matrices, scaffolds) that is analogous to the skin extracellular matrix [20, 21]. Artificial skin equivalents are films made of various organic polymers that protect the internal environment of the body from the external environment when the skin is damaged, allowing regeneration of the damaged skin; they are not full skin substitutes [22]. Living skin component equivalents (LSCEs) constitute a three-dimensional organ culture consisting either of a cellular component only [23, 24] or of a cellular component with a biopolymeric matrix that mimics one, two, or more layers of the skin [25–30]. This review uses LSCEs to refer to both single- and multilayered equivalents, as well as skin derivative equivalents, and LSEs to refer to equivalents that contain at least dermis and epidermis analogs. Modern models of LSEs are most often modifications of the classical bilayered version, which mimics the dermis and epidermis, or they represent a fundamentally different model. They can have a more complex structure and include skin derivatives such as the HFs and sebaceous glands [31].

## 4. Types of Skin Equivalents

No single classification of LSCEs includes all types of models. However, it is possible to group all types of equivalents according to the number of components in their composition into one-component (primitive equivalent of derma or epidermis only) [23, 24, 32], bicomponent (primitive equivalent of dermis and epidermis, often called full-thickness LSEs) [25, 26], three-component (equivalent of dermis, epidermis, and hypodermis) [27], and multicomponent (several cell subpopulations within an LSE) [33]. It is also conventionally possible to distinguish cutaneous organoids as a separate type of inverted spherical-shaped LSEs [34, 35], as well as organoids that are follicle-like structures [36, 37]. LSEs that include the equivalent of the epidermis can be further subdivided into keratinized equivalents if epithelial cells form a corneal layer during cultivation and nonkeratinized ones if they do not. Vascularized LSEs [2] are distinguished from LSEs containing skin derivatives (HFs or follicle-like structures, or sebaceous glands) [29, 38].

## 5. Demand for Ideal LSCEs and Their Applications

The main objectives of techniques based on LSCE transplants include protection of the skin from dehydration and environmental factors, such as pathogenic microorganisms, immediately after injury, and functional compensation of the damaged skin fragment (LSCE use is not required in cases of epidermal damage only) [39]. LSCEs can be used to replace skin fragments or the entire skin in cases of genetically determined developmental defects affecting the skin and/or skin derivatives, where these mutations lead to a disruption of the patient's normal life activity [40].

Skin derivatives obtained in vitro can be used for alopecia therapy with lab-generated HFs [41]. LSCEs are used for pharmacological and cosmetic purposes as relatively representative and bioethical models for testing various medications and cosmetic preparations, as well as for studying the toxicity of various factors [33, 42]. LSCEs can be used to study the biotoxic and pathophysiological effects of bacteria [38]. The development of LSCEs under in vitro conditions can help to study the morphogenesis of both the skin and its derivatives, as well as to study skin regeneration processes since some models of LSCE production demonstrate most of the stages and signaling cascades that occur during the formation of the skin and its derivatives in the course of embryogenesis [31, 41]. Thus, the development of technologies for generating LSCEs similar to their native counterparts is an important task for modern translational medicine, as well as for research on the morphogenesis of the skin and its derivatives, pharmaceutics, and cosmetology.

Full-thickness LSE can be used for skin remodeling and regeneration after keloid removal. Scores from the Patient Scar Assessment Scale (pain, itch, color, stiffness, thickness, and irregularity) showed a trend towards improvement in most participants, with particular improvement in itchiness [43].

## 6. Requirements for an Ideal LSCE Within a Wide Range of Tasks

The ideal LSCE must have several qualities to ensure its universal application to a wide range of demands:

1. Potential for effective transplantation. This implies either successful integration of the graft at the site of the damaged skin area with stimulation of regeneration and generation of a temporary protective barrier and subsequent replacement of the graft tissue by the recipient skin, or prolonged integrity of the graft and maintenance of its cellular and extracellular composition after transplantation in substitution therapy. To meet this criterion, the graft must be hypoallergenic with respect to the recipient's immune system, ensuring that the graft will not be rejected; this requirement is a prerequisite for successful integration of the graft into the recipient's body. This point is especially important when testing LSCEs with allogenic components [39, 44]. Autologous, allogeneic, and hybrid (containing both autologous and allogeneic components) LSCEs are suitable for medical purposes by this criterion [24].
2. Safety of transplantation. For any medical graft, sterility and absence of uncontrolled genetic modifications are essential. Any interaction of underlying tissues with the graft should be accompanied by an integration of the blood and nervous systems of the recipient with LSCEs and should not lead to an initiation of tumor growth [39, 44, 45]. This point is most relevant within the medical application of LSCEs.
3. Nativity. LSCEs and skin derivatives should be as close as possible to their native counterparts in their macro- and microstructure, cellular composition, and physiology, including extracellular matrix and lipid composition of corneal envelopes of the epidermis. An ideal LSE should contain functioning analogs of the dermis, epidermis, hypodermis, and skin derivatives, including HFs, sweat glands, and sebaceous glands [39, 41, 44, 45].
4. Cost effectiveness. The time and monetary costs required to generate equivalents should be minimized. Moderately priced LSCEs should be available for medical, scientific, and pharmacological purposes [39, 44]. Allogeneic LSCEs are suitable for widespread use.
5. The possibility of cryopreservation. LSCEs should retain their composition and structure during cryopreservation and long-term storage in this state. The possibility of cryopreservation allows the use of ready-made LSCEs or functional units, from which LSCEs can be prepared in a short time, immediately when the need arises; this is critical for both pharmacological tests and translational medicine. This requirement imposes certain limitations on the physical size of LSCEs since the diffusion of the cryoprotectant across the graft can be uneven and prolonged, leading to cytotoxic effects or insufficient protection of the equivalent from liquid crystallization in cells [46, 47].

It seems that the requirements for ideal model LSCEs and LSEs suitable for translational medicine are to some extent incompatible with each other. However, the development of technologies and models for constructing LSCEs to meet the above requirements continues.

## 7. Evolution of LSCE Models and Skin Derivative Models in 20th Century

### 7.1. The First Model: Single-Component Equivalents of the Dermis and Epithelium

**Figure 1:**
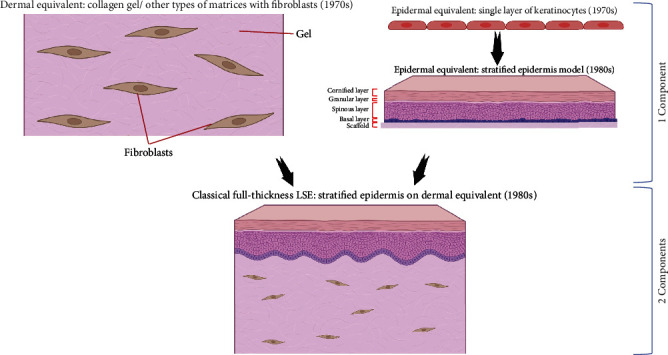
Evolution of living skin component equivalent (LSCE) models in the second half of the 20th century. Generation of classical full-thickness LSE from one-component LSCEs. The figures were made using the online editor BioRender.

The prospect of using primary human skin cells or their analogs to generate LSCEs appeared simultaneously with the development of tissue engineering, in the 1970s. The first significant step before the invention of the technology of LSCE generation was the development of a method for the isolation and cultivation of human dermal fibroblasts from skin fragments [48]. The first dermal equivalent model based on the use of collagen gel as a carrier was described by Karasek and Charlton in 1971 [49] and was further developed and implemented by Bell, Ivarsson, and Merrill in 1979 [32]. This model utilized a mixture of collagen, primary human fibroblasts, and other components to form a dermal equivalent based on collagen gel (Figure 1). With polymerization, this formed a dermal equivalent of the human skin. The thickness and density of this skin equivalent depend on the number of cells used, the concentration of collagen in the solution, and the height of the vessel in which the polymerization takes place. The disadvantages of this model include low proliferative activity and self-maintenance of fibroblasts in the system, due to the absence of many native signaling systems present in the dermis of normal skin; compression of the collagen gel, due to the contractile activity of fibroblasts; and destruction of the collagen gel by matrix metalloproteases synthesized by them [50]. In addition, when this type of LSCE is used in translational medicine, epithelialization of such equivalents to replace extensive skin lesions occurs only after 3 weeks after transplantation [51].

The epithelial equivalent skin model was established in 1975 after the technology of isolation and cultivation of keratinocytes was developed by Rheinwatd and Green [52]. O'Connor et al. were the first to use sheets of primary keratinocytes, grown in vitro from autologous keratinocytes, for transplantation to two patients with extensive burns [23]. In this model, human keratinocytes are placed on Vaseline gauze, forming an analog of the basal layer, which can then form an analog of the stratified epidermis with formation of the spinous and corneal layers after grafting (Figure 1). A liquid-air interface at the border of the basal and overlying layers is needed to form a stratified epidermal equivalent. Among the main disadvantages of such equivalents are their low viability and poor engraftment, mainly at the site of wounds or burns with severely damaged dermis. Stratification of the epithelial equivalent presents certain difficulties [53–55], which, to some extent, are not important, since keratinization can be restored after transplantation, but do reduce the immediate protective properties of the graft. This first, albeit somewhat primitive, tissue equivalent initiated the development of the field of tissue engineering, which half a century later is regarded as one of the most promising and booming fields of medicine of the 21st century.

### 7.2. The Classical Bilayered LSE Model

The second stage in the development of LSEs, described in the early 1980s, was the combination of dermis and epidermis equivalents to create two-component LSEs (Figure 1) [56]. Most technologies for this type of LSEs were based on a two-component model (LSEs with epidermal and dermal equivalents) established on Type I collagen gel. Other carriers, in particular, collagen sponges and various multicomponent scaffolds, can be used instead of collagen gel. A promising carrier is a collagen sponge consisting of sodium alginate and fibrinogen with the inclusion of thrombin [57, 58]. The search for the ideal carrier is ongoing with the aims of preventing biodegradation of the organic matrix during the activity of the cellular component in the LSEs and providing optimal conditions for maintaining cell viability for the maximum amount of time.

Classical models allow generation of LSEs in several stages in a time frame from 2 weeks to 1 month, depending on the protocols used. A method to grow fibroblasts in collagen gel on special membranes impermeable to cells but permeable to the medium (in systems like Transwell^R^) facilitated medium replacement in the system, increased the maximum volume of the medium, and allowed the growth of LSEs with a liquid-air interface at their surface, which is necessary for obtaining a stratified epidermis [56]. To achieve this, keratinocytes are placed atop the collagen gel and placed in direct contact with the air at the final stage, while the dermal layer is submerged in a culture medium with increased calcium ion concentration [56]. Providing direct contact between the epidermal part of the LSE and the air and introducing an increased concentration of calcium and chlorine stimulate the stratification of the epidermal layer and also promote an intensive synthesis of the matrix by the cells of the tissue-engineered construct.

The conditions provided in this model lead to formation of an analog of the basement lamina between the fibroblasts and the keratinocytes. At the final stage of LSE generation according to this model, the presence of the following immunohistochemical markers at various levels of the LSE can be recorded: Collagen III through the entire dermis, Collagen VII at the level of the basement membrane, IV at the dermis and basement membrane, K14 in the epidermal basal layer analog, K1 in the spinous layer analog, and involucrin in the stratum corneum [25, 26].

Despite its prevalence, the classical LSE model, including a dermal equivalent made of an organic matrix with fibroblasts and an epidermal equivalent of several layers of keratinocytes, had several drawbacks. Resulting from interaction between fibroblasts and keratinocytes in a three-dimensional system, the bilayered LSE structure was similar to a fragment of dermis and epidermis of the skin, but without vessels, nerve endings, receptors, and skin derivatives and also without a stratification of the dermis into reticular and papillary sections. For 30 years, primary human skin cultures, which are autologous only in relation to their donor and have limited proliferative potential, served as a source of cellular material for LSEs. Metabolic changes in the burn victim's body may increase the difficulty of cultivating autologous cells for LSEs for burn treatment. In addition, it takes a considerable time from the moment of biopsy to LSE transplantation to manufacture LSE for a large area, reducing the effectiveness of this approach. Other disadvantages of this technology include the dependence of cell viability and proliferation on the age of the donor [59, 60] and on several other factors, as well as the absence of many physiological properties inherent to native skin. All the facts described above have severely limited the use of classical LSEs both for clinical purposes and as a model for testing various types of exposures [61, 62].

## 8. Current Commercial LSCEs

Many currently available commercial dermal equivalents have passed preclinical and clinical trials [63–66]. Most modern biocompatible dermal grafts have mechanical properties close to those of human dermis. They provide structural integrity and elasticity and are successfully used in the treatment of burns, ulcers, wounds, and chronic diseases. Some commercial LSCEs are optimized for pharmacological and scientific tests. Most are able to generate stratified epidermis with stratum corneum [42]. Allogeneic or autologous fibroblasts and keratinocytes are used as cellular material in all models. Collagen gel, collagen sponge, hyaluronic acid, or hydrogels of artificial origin can be used as a carrier [63–66]. Epidermal equivalents grown from patient cells are also used [67, 68]. Some commercial LSCEs for clinical use are noted in Table 1; Table 2 lists some commercial LSEs for research and pharmacology tests.

There are also commercial full-thickness LSEs, usually based on collagen gel–based scaffolds, collagen sponge, or other types of carriers using both allogeneic and autologous fibroblasts and keratinocytes. Cells isolated from the foreskin of newborns are often used as allogeneic material [69, 70]. The use of these products is most justified for extensive and severe skin lesions, as well as to avoid the formation of connective tissue scars at the site of lesions [45].

## 9. Alternative Approaches to Obtaining Ideal LSCEs

These methods of LSCE generation were based on a technology that is expensive, in comparison with synthetic polymeric analogs (within the framework of single-copy generation). This technology involves labor-intensive manual operations with cell lines isolated from the human skin, which does not allow producing significant amounts of LSCEs quickly and limits application in translational medicine and pharmacology. This method may have problems with the reproducibility of the procedure. In addition, previously obtained full-length samples are difficult to cultivate in a conventional CO_2_ incubator due to problems with saturation and metabolism in the central region of the equivalents.

**Figure 2:**
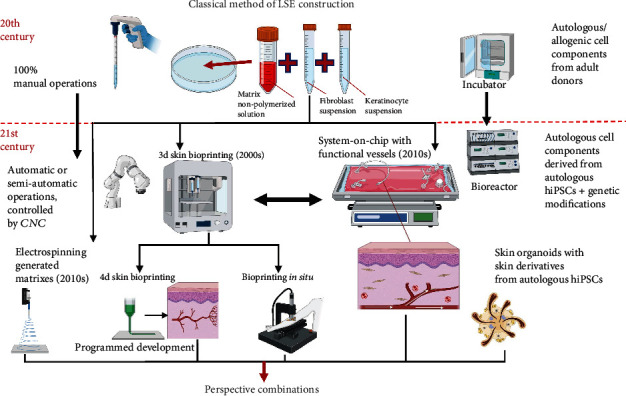
Evolution of methods for obtaining LSEs and the prospects of combining the latest developments in this area. CNC: computer numerical control, hiPSCs: human-induced pluripotent stem cells. The figures were made using the online editor BioRender.

**Figure 3:**
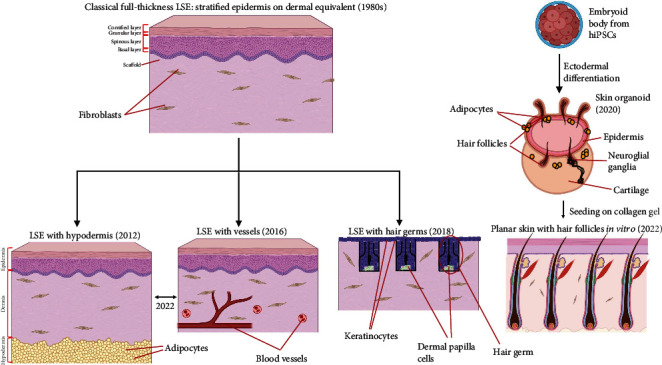
Emergence of new modifications of the classical LSE (left) and a fundamentally new model of skin organoids (right) in the 21st century. The figures were made using the online editor BioRender.

The imperfections of these technologies for obtaining LSCEs have led to early 21st-century attempts to improve them. One of the distinctive features of the new approaches is the transition from manual operations with cells to automatic systems providing both the cultivation of cell lines and equivalents (transition from incubators to bioreactors and systems on a chip) and the generation of the equivalents themselves [71–73] (Figure 2). The new methods have been designed to increase accessibility and generation speed, to bring LSCEs closer to their native counterparts, and to expand their applications. New approaches to LSCE generation include generation of LSEs from cells obtained during differentiation of pluripotent cells, obtaining LSEs using 3D and 4D printing, developing skin organoids, which can be used for LSE generation during differentiation of embryoid bodies, and LSE generation in microfluidic systems. New models allow obtaining LSEs that are morphologically and functionally closer to the native skin (Figure 3). New modifications of the classical LSE model include full-layer three-component LSEs with hypodermis, vascularized LSEs, and LSEs with HF buds. LSEs are being developed with skin derivatives: sebaceous glands, smooth muscle cells, adipocytes, and several other cell lines derived from skin organoids placed on collagen gel.

### 9.1. Alternative Cell Sources

Attempts have been made to solve the problem of the limited potential of using primary cell lines for the generation of LSEs by employing their analogs, cells obtained in the course of differentiation of pluripotent cells. iPSCs, analogs of embryonic stem cells (ESCs), were first obtained by a team led by Takahashi et al. in 2007 [74]. The use of iPSCs, as a more bioethically acceptable, convenient, and inexpensive resource for obtaining mammalian somatic cell lines, compared with the use of human embryonic cells derived from the inner mass of the blastocyst in embryogenesis, has significantly accelerated this direction of translational medicine. Now it is possible to use autologous lines of cells, phenotypically close to the cells isolated from embryonic or postnatal tissues, to create LSEs for the potential donor.

With the advent of technologies to obtain hiPSC, protocols for differentiating or reprogramming hiPSCs into fibroblasts [75], keratinocytes [76, 77], dermal papilla cells [78, 79], melanocytes [80], or adipocytes [81] have been developed. Attempts to form bilayered LSEs based on the classical model have used fibroblasts and keratinocytes derived from hiPSC [82, 83]. The LSEs formed in this way are not fundamentally different from the previously obtained classical models based on primary cell lines, but the cellular material for their production is significantly more accessible. Kim et al. [82] generated full-thickness LSEs from hiPSC-derived KCs and FBs and successfully implanted them into immunodeficient mice, maintaining them for 2 weeks. The factors for epidermal differentiation were BMP4, retinoic acid, and EGF. The factors for dermal differentiation were BMP4, insulin, and FBS. No visible tumors, scars, or keloids were detected. The LSE was formed on membrane inserts based on Collagen I gel mixed with hiPSC-derived fibroblasts and covered by hiPSC-derived keratinocytes. This skin graft demonstrated a morphology similar to that of real skin. In 2013, an organoid with an epithelium-lined cyst in the center with HFs was generated from mouse embryonic keratinocytes and fibroblasts cultured in the presence of 2% Matrigel®. Complete HFs 3 mm long, with shafts oriented inside the cyst's cavity, formed within 1 month. This folliculogenesis required no expensive growth factors for induction. Disadvantages of this approach include the difficulty in obtaining initial cells and the short interval of initial cell line cultivation during which folliculogenesis will be effective [84]. It has not been possible to obtain fully functional HFs with human cells in this way, due to the unethical nature of such procedures. In 2019, another research group showed that iPSC-derived CD200^+^/ITGA6^+^ epithelial stem cells displayed the phenotypes of HF stem cells. The differentiation factors were BMP4, bFGF, FBS, insulin, hydrocortisone, cholera toxin, triiodothyronine, and retinoic acid. After seeding on the human amniotic membrane, iPSC-derived epithelial stem cells proliferated and demonstrated stratification. After transplantation into mouse skin wound, CD200^+^/ITGA6^+^ epithelial stem cells on the human amniotic membrane promoted the construction of HFs and interfollicular epidermis, without any tumors or keloid formation [85]. There is a potential for similar technologies in obtaining cells with the phenotype of embryonic fibroblasts and keratinocytes during directed differentiation of hiPSCs or genetic modification of postnatal cells.

### 9.2. New Scaffolds for LSECs

A variety of materials are used as scaffolds or substrates for LSCEs, providing structural support to tissues and imitating the extracellular matrix, supporting cell adhesion, proliferation, and differentiation. The scaffold determines the structural and physiological properties of the dermis analog, determines epithelial cell adhesion, and creates a niche for fibroblasts and basal keratinocytes. Therefore, the choice of matrix material is one of the key parameters that determine the successful use of developed LSCEs for tissue engineering and regenerative medicine. Both natural-derived substances and synthetic polymers can be used as materials, and both types have their advantages and disadvantages.

Synthetics used to generate LSCEs include degradable polyglycolides, polylactides, polylactide-coglycolides, polycaprolactones, and polypropylenes; the most widely used nondegradable matrix is polyurethane [86]. The use of synthetic materials makes it possible to precisely control the matrix composition and obtain structures with specified mechanical and chemical properties, specific structure, and desired size [87]. Synthetic matrices are usually inert, do not undergo contraction, and retain their original structural properties during long-term cultivation of LSCEs and after transplantation. The main disadvantages of synthetic materials are the lack of chemical signals for cell recognition and adhesion and lower biocompatibility. For this reason, synthetic polymers are used mainly in combination with various natural substances, most commonly collagen [88]. For example, the combination of polycaprolactone and collagen enhances cell proliferation within the scaffold and promotes faster wound closure and accelerated regeneration in vivo [89, 90]. The use of scaffolds made of poly-(L-lactic acid) modified with collagen results in enhanced cell adhesion and migration and subsequent formation of epidermal layers on the surface of the construct [91]. Composite materials are also used as a matrix for commercially available products. These include Transcyte®, which consists of a nylon mesh embedded in a silicone layer coated on top of a collagen gel with neonatal fibroblasts, and Dermagraft®, which consists of human fibroblasts grown on a biodegradable polyglactin mesh [87].

For the fabrication of commercial skin equivalents and their use in clinical practice, natural biomaterials are preferred because of their low toxicity, minimal inflammatory response, and similarity to native skin matrix [92]. However, natural polymers in pure form usually have low structural stability and mechanical properties, which may contribute to graft wound contraction and scar formation [86]. To improve biostability, natural polymers are often chemically or physically modified, combined with other biopolymers such as glycosaminoglycans, fibronectin, and chitosan, or with more mechanically stable synthetic polymers [86, 87].

One of the most widely used natural materials is collagen, the main protein of the extracellular matrix of the dermal layer. The main advantages of this biomaterial include its relative availability, biodegradability, biocompatibility, and ability to modulate cell behavior and accelerate wound healing [93]. Collagen-based matrices are currently used both to create classical bilayer LSCEs, including commercially available products, and to produce more advanced skin models incorporating skin derivatives [83, 93, 94].

A number of studies have used collagen in gel form as a matrix to produce three-dimensional equivalents of skin and HFs, by promoting cell adhesion and self-organization [29, 95]. Collagen hydrogel is also widely used as a biomaterial in bioprinting technology [96, 97]. The main advantage of these types of matrices is their high biocompatibility due to their similarity with the macromolecular components of tissue, as well as their controlled biodegradation rate [98]. However, the mechanical properties of collagen hydrogel produce compression due to the contractile activity of fibroblasts; this may hamper culturing of the LSCEs and reduce the positive effect of its engraftment after transplantation [50]. In addition, the organization of collagen fibers can be influenced by external factors such as changes in pH and temperature, which can lead to heterogeneous matrix structure and affect intercellular interactions and cell-matrix interactions [99]. It is possible to partially overcome these limitations by crosslinking collagen. Chemical crosslinking of the collagen gel has been shown to improve mechanical stability and avoid reduction of the scaffold area during culturing while not affecting cell viability and differentiation [100]. In addition, the mechanical and physiological properties of collagen gel are determined by its density. Higher collagen density in hydrogel has been shown to reduce collagen contraction and improve stability during prolonged culturing compared to gels with lower concentrations and to reduce proliferative and contractile activity of fibroblasts [101, 102]. To increase the biostability of matrices, modulate cell behavior, and improve healing, various modifications of collagen have been developed by combining it with other synthetic or natural polymers [86, 103]. For example, a composite scaffold based on Type I collagen and tropoelastin has stimulated proliferation and migration of dermal fibroblasts in vitro, and at transplantation supported fibroblast infiltration, de novo collagen deposition, and neovasculogenesis [104].

Another “natural” matrix is Matrigel®, which consists mainly of laminin and Collagen IV that form a three-dimensional structure during polymerization. It is often used for three-dimensional cell culture and organoid generation [105]. Such a matrix helps to recreate the native skin microenvironment, which promotes cell self-organization and influences cell differentiation. A Matrigel®-based culture system has been successfully used to generate dermal papilla spheroids and skin organoids with HFs from iPSCs [34, 35, 106, 107]. However, Matrigel® may have significant variability in composition from batch to batch, including the presence of xenogeneic proteins, which in theory may influence cell behavior and therefore the reproducibility of results [108]. In addition, Matrigel® is derived from mouse tumor cells, potentially limiting its use in clinical practice due to immunogenicity.

A number of other natural polymers such as fibrin, chitosan, and hyaluronic acid, although much less common, are used as matrices for generating LSCEs, mainly after chemical modifications or in combination with other biomaterials. For example, modified hyaluronic acid–based scaffolds are now successfully used for the generation and clinical application of LSCEs, and hyaluronic acid–based hydrogels are used for bioprinting [98, 109]. Decellularized human, porcine, or bovine dermal extracellular matrix is often used as a natural scaffold material [110]. It is as close to native tissue as possible, as it preserves the complex composition of the extracellular matrix and the natural architecture of the dermis, providing an optimal microenvironment for cell adhesion and proliferation [110, 111]. However, this type of scaffold has several limitations such as poor mechanical properties, difficulty in standardization and manipulation in culture, and risk of infection during transplantation.

Another promising approach to generating LSCEs is the use of plant-derived scaffolds, which have greater biocompatibility than animal-derived ones and significantly reduce the risk of transmission of pathogens or development of an allergic reaction and inflammation; this makes them preferable for use in regenerative medicine [112]. Creation of recombinant human proteins from plant material has produced plant-derived human collagen (PDHC), from which two types of tissue-engineered scaffolds have been generated. Despite their lower mechanical properties, PDHC scaffolds have successfully supported the attachment, proliferation, and viability of primary fibroblasts and keratinocytes, leading to the formation of a stratified keratinizing skin epidermis [113]. One research group has generated a soy protein–based scaffold for primary fibroblasts of the human skin. Such scaffolds support the adhesion and proliferation of cultured fibroblasts. Cells attached to soy scaffolds or to classical collagen scaffolds of animal origin express similar sets of extracellular matrix proteins, integrins, and metalloproteinases [114]. Plant proteins are increasingly utilized in the fabrication of tissue engineering scaffolds, although their clinical applications are still limited [115]. Plant-based matrices are unlikely to be suitable for generating complex multicomponent LSCEs containing skin derivatives, as substances closer to the native extracellular matrix are preferred in this field.

A number of bioorganic and synthetic materials are used to form a substrate for keratinocytes when creating single-layer LSCEs, analogs of the epidermis. These can be components of the skin basement membrane (Collagen I, Collagen IV, laminin, fibronectin, or combinations), Matrigel®, or decellularized matrix from amniotic membrane or from cultured fibroblasts [116–119]. A promising substrate is spider silk fibroin, which promotes quality adhesion and proliferation of keratinocytes [120]. A recent study has demonstrated that a decellularized matrix allows the formation of an equivalent of epidermis with regular morphology of each of the epidermal layers and with epithelial cells forming hemidesmosomes. Among synthetic substrates, polycaprolactone is a promising option [121]. It has recently been shown that the keratinizing equivalent of the epidermis can grow on a substrate of poly(N,N′-hexamethyleneadipinediamide) (N6/6) manufactured by electrospinning [122]. Bioorganic and synthetic substrates of epidermal LSCEs are characterized by similar advantages and disadvantages as the matrices of full-layered LSCEs.

Thus, matrices and substrates based on bioorganic components are more suitable for the creation of LSCEs for pharmacological tests, research, and replacement therapy due to better reproduction of native skin properties. However, synthetic matrices may be less expensive and more convenient for creating commercial LSCEs that are used for temporary wound coverage.

### 9.3. The New Technology of Scaffold Formation

A new approach to improving the mechanical properties of the collagen matrix is the creation of three-dimensional collagen scaffolds using various extrusion methods, electrochemical methods, gas foaming, exposure to a strong magnetic field, freeze-drying, electrospinning, solution blow spinning, mechanical methods, or a combination of these methods [103, 123–125]. Technologies for producing scaffolds by electrospinning and lyophilization are widespread and under active development. Freeze-drying is a relatively fast and affordable approach to produce scaffolds with a porous structure due to sublimation of water solution after freezing that provides suitable conditions for cell seeding, cell infiltration, and extracellular matrix production. However, freeze-dried constructs often exhibit heterogeneous pore size and structure, which can prevent uniform distribution of cells [126, 127]. Electrospinning is a process of solid fiber (composed of a viscous polymer solution) production by the generated electrical field. Electrospun scaffolds have a more homogeneous collagen organization and smaller pore size, and this microporous architecture is the closest to the native fibrillar structure of the extracellular matrix, which creates optimal conditions for cell adhesion, proliferation, and differentiation [128, 129]. When electrospun and freeze-dried scaffolds are compared as a matrix to create a dermal equivalent, no significant differences are found in cell proliferation, organization, and viability, or in their ability to differentiate into stratified epidermis. However, after transplantation, electrospun scaffold demonstrated less wound contraction and more efficient healing compared to the freeze-dried construct [103, 126]. Some other advantages of electrospinning include the possibility of small pore size that can prevent microbial penetration, and the possibility of incorporating various bioactive compounds, for example, growth factors or therapeutic agents, into electrospun nanofibres to improve biofunctional properties as well as drug delivery to improve wound healing [130]. An important advantage of electrospinning and similar technologies is their compatibility with 3D bioprinting, making it possible to generate LSEs with scaffolds on hydrogel and fibers with improved mechanical properties and to generate blood vessel networks from fibers in resulting constructs [131].

### 9.4. Three-Dimensional Bioprinting

Bioprinting is an advanced high-throughput method based on traditional 3D printing that allows the automated production of a target sample based on 3D models with a high degree of accuracy by layer-by-layer application of biomaterials, living cells, and growth factors [96, 132]. Thus, 3D bioprinting is an additive technology where biomaterials and living cells are used as the applied material that forms the sample layer-by-layer, forming the equivalent of an organ or tissue [133]. The concept of bioprinting, including the use of hydrogels and cells as bioinks, was developed and applied in the 2000s by the teams of Mironov et al., Pardo, Cris Wilson, and Boland, and Odde and Renn [134–136]. The first LSE models obtained using 3D bioprinting came later [137, 138]. The use of 3D bioprinting technologies makes it possible to form deterministic patterns of cell subpopulation distribution in the formed structure so that during modulated morphogenesis, these subpopulations provide, through their interactions, during the maturation of the resulting equivalent, the formation of certain structures, such as the dermis, HFs, or blood vessels. Another advantage of using additive technologies is the speed of making a dermal equivalent of a given thickness and shape using layer-by-layer bioprinting compared to the speed of making a classical collagen gel [96]. It is possible to apply both individual cells and aggregates (spheroids) of these cells, as well as various organic polymers, in any combination with each other [139]. In the last few years, the development of additive technologies of 3D bioprinting, the advent of cell differentiation technologies, and increased knowledge about organ morphogenesis have led to the design of models for obtaining LSEs with integrated HFs and blood vessels using somatic cells and hydrogels of different types. One of the topical directions of 3D bioprinting is the use of endothelial cells, which can provide vascularization of the formed 3D equivalent [140].

Currently, skin bioprinting is performed using three main methods: extrusion bioprinting, microvalve bioprinting, and laser-induced direct transfer printing [96, 141]. Extrusion bioprinting, in which cell suspensions and hydrogel are fed to the substrate using an extruder in the form of a jet, is the cheapest and easiest method of bioprinting. Extrusion printing is the fastest method, but also the least accurate one, since it does not accurately reproduce the complex patterns of cell and extracellular matrix distribution within a single layer [142]. Microvalve bioprinting involves separate reservoirs of cell suspensions and unpolymerized hydrogels, which are discretely fed to the substrate in the form of droplets using separate valves. The advantages of this approach are good homogeneity, printing speed, and a wide range of final scaffold viscosity. Disadvantages include problems with extruder nozzle clogging, less stable homogeneity of the applied material, and lower maximum cell density, compared with laser-mediated 3D printing [137, 143, 144]. Laser-mediated printing of LSEs occurs by transferring droplets of partially polymerized hydrogel mixed with cells from the laser-activated area of the donor slide to the underlying substrate. This method gives good accuracy and resolution of bioprinting, a slightly better cell survival in the formed LSEs, and a higher cell density, compared with the previous method. The disadvantages of laser-mediated bioprinting are limitations of the viscosity range of the hydrogel used, a lower homogeneity of the formed structure, problems with rapid drying of the hydrogel, the risk of foreign cytotoxic particle transfer from the donor slide, and a high cost [145–148]. Collagen, decellularized extracellular matrix, fibrinogen, cellulose, pectin, gelatin, hyaluronic acid, and some other biopolymers are commonly used as hydrogels [96, 141].

A recent bioprinting study produced a vascularized bilayered LSE [97]. A dermal analog was constructed by coapplication of dermal fibroblasts, vascular endothelial cells, placental pericytes, and Type I collagen. Human keratinocytes were applied as a second layer. The formed LSEs contained not only the dermis and epidermis analogs but also blood vessels. Four weeks after the transplantation of this LSE into immunodeficient mice, the blood vessels that had been formed de novo were integrated into the animals' circulatory systems. In the future, producing LSEs using this technology will make it possible to obtain LSEs that will not be replaced by the host's own cells for a long time and potentially will be used in the treatment of many skin diseases and to achieve recovery of the skin after deep burns [97]. A further example of the use of 3D printing is the generation of a model of HF and sweat gland interaction by recreating a 3D system consisting of mesenchymal cells and a matrix that mimics the sweat gland and then integrating HF germ spheroids into this matrix [149].

Another promising and unique direction in the field of 3D skin bioprinting is in situ bioprinting. This method of skin bioprinting forms an LSE directly in the area of skin damage on the patient's body, in case there is no time to form an LSE in a given situation such as an extensive skin burn [150]. This type of printing requires both the development of a special type of hydrogel capable of fast and efficient attachment to the damaged area on the patient's body and modification of the printing platform itself: in particular, the possibility of three-dimensional articulating movement of the movable bioprinting head with several degrees of freedom. The problem of compatibility of the new type of bioprinting with cellular material placed in hydrogels remains relevant.

3D skin bioprinting in its classical meaning still has several limitations associated with its inability to reproduce a fully functional skin structure with its derivatives in an in vitro system, since in its current version, bioprinting does not reproduce the conditions for the diversity of different cell subpopulations and skin appendages in the generated structure. However, bioprinting has the potential to scale up and simplify generation of LSEs of different types [96]. The main promising advantages of applying bioprinting in the medical industry are the possibility of reducing manual operations, the prospect of significantly scaling up, and standardizing LSE production, which should potentially reduce the cost of ready-made therapeutic solutions.

### 9.5. Microfluidic Systems

Formed full-thickness LSEs several millimeters thick have problems with gas exchange, nutrient intake, and excretion of metabolic products due to physical limitations in these processes. The solution is to create a network of branched vessels made of hydrogel or endothelial cells inside the dermal component of the LSE, imitating the connective tissue vessels, and providing a directed liquid flow through these vessels. One of the goals of microfluidics within tissue engineering is to design systems that support and control the passage of the nutrient medium through the organoid chamber, as well as control the composition and properties of this medium. Modern microfluidic systems can perform a gradual replacement of the medium, as well as monitor and maintain its pH, carbon dioxide and oxygen levels, ion content, and temperature [151]. A successfully tested system on a chip is an example of the formation of a complete vascularized two-component keratinized LSE based on a microfluidic system. In this study, the advantage of the microfluidic system is evident in the quality of microvascular bed formation, the correct morphology and thickness of the dermal and LSE components, and the expression of markers of terminal epidermal differentiation [72]. All of these strategies can be used in combination with each other to increase the efficiency of obtaining LSEs with given properties and can also increase the time during which the finished LSE retains its viability [152].

### 9.6. Prospects of Four-Dimensional Printing and the Integrated Approach

In 2012, Tibbits described the concept of using materials with the inherent potential to change their structure according to a specific pattern under an external stimulus, and in 2014, he introduced the term 4D bioprinting, defining it as a technology that allows the generation of a 3D structure from living cells capable of deterministic development under the influence of external or internal stimuli according to patterns programmed by the operator and/or capable of changing structure over time [153, 154]. In practice, this technology has not yet been fully applied to LSE bioprinting. However, it is possible to name options for the application of various 4D bioprinting approaches to this field of translational medicine [155]. An example of the use of this technology is the formation of self-assembled tubes from hydrogel of a specified composition, which mimic the smallest blood capillaries [156]. Bioprinting of self-assembled hybrid hydrogel structures with the inclusion of various growth factors and cytokines can in theory be used to form a guided network both for vascularization and angiogenesis and for the directed growth of neuronal axons in LSEs or living equivalents of other organs after the implantation of the printed structure into the recipient's body. This approach makes it possible to first scan the unique vascular pattern of the patient's skin, obtain a three-dimensional model, and then provide, at the level of the primary printed sample, further layering of this vascular pattern during LSE maturation. In addition, it is possible to create collagen gels with wells for the transplantation of HF germs with a determined angle of hair growth. Nevertheless, this approach does not guarantee the reproduction of all conditions that would ensure the formation of an LSE and its derivatives during embryogenesis. Additionally, this method requires, on the one hand, a full understanding of all mechanisms of cell differentiation present in normal skin and its derivatives and, on the other hand, a complete grasp of the mechanisms of interaction of all cell subpopulations with each other and with their microenvironment within these structures [157].

## 10. Modern Modifications of LSCE Models

### 10.1. Vascularized Classical LSEs

The first vascularized LSEs appeared in the 2010s [140]. This is another superstructure of the classical LSE model, which comprises introduction of endothelial progenitor cells together with fibroblasts into collagen gel or another carrier. As a result, a microvascular bed is formed in the dermal analog. In 2016, for the first time, it was possible to create a full-thickness LSE with capillaries. This study used primary fibroblasts, primary endothelial cells of the microvascular bed, and primary human keratinocytes in a commercial polymeric scaffold. By coculturing this system in a bioreactor, it was possible to form a keratinized epithelium and dermis in which angiogenesis occurred with the subsequent formation of a branched capillary network [28]. This improvement allows improved gas exchange, nutrient supply, and removal of metabolic products throughout the volume of the LSE, preventing the formation of a necrotic core, and also allows creation of a more representative LSE model for dermatological studies.

### 10.2. Three-Layered LSEs

Attempts to create a three-layered LSE model using adipocytes to mimic the hypodermis were previously unsuccessful because a simple combination of postnatal cell layers, in the absence of a nervous and circulatory system, did not facilitate proper signaling between the cells. The increased thickness of the LSE with the additional layer complicated the supply of nutrients and oxygen to the cells in the central area of the equivalent. This resulted in failure of formation of a complete LSE, and the epidermis, dermis, and hypodermis analogs did not maintain full contact with each other [27].

Later attempts to create a more advanced LSE with the inclusion of adipocytes were more successful [158]. Adipocytes were placed on a collagen substrate, and a layer mimicking the hypodermis was built up. At the second stage, a vascularized dermal layer was created using endothelial stem cells and fibroblasts, and the hypodermal layer was vascularized in parallel. At the third stage, keratinocytes were added and the resulting epithelial layer was placed into a liquid-air interface, providing stratification and formation of a multilayered epidermis. This produced a LSE as a complete integral three-layered structure, capable of self-sustaining due to the formed vessels [158]. There is evidence that the hypodermis plays an important role in wound healing [159], and the three-layered vascularized LSE has a potential advantage over its counterparts in medical applications.

### 10.3. LSEs With Follicle-Like Structures

Another promising model created in recent years demonstrates the possibility of obtaining LSEs with integrated immature HF in vitro. This technology involves placing dermal papilla cells and keratinocytes in conditions that mimic conditions that trigger folliculogenesis. In one of the first studies using this approach, molds with plastic rods were made on a 3D printer. The dermal layer of the future LSE was then produced by filling these molds with collagen gel and fibroblasts. The molds were removed, and dermal papilla cells genetically modified to overexpress Lymphoid Enhancer-Binding Factor 1 (LEF1) were placed into the formed wells, followed by a suspension of keratinocytes. Overexpression of LEF1 in postnatal dermal papilla cells stimulated their epithelial-mesenchymal interactions with keratinocytes. With selected culture media, the interaction between keratinocytes and dermal papilla cells stimulated folliculogenesis with the formation of follicle-like structures. However, hair formation occurred only after the transplantation of the LSEs into the skin of immunodeficient mice [29]. No visible tumors, scars, or keloids were detected.

Similar results were achieved using mouse dermal papilla cells and outer root sheath cells isolated from HF. They were used to form organoids in which epithelial-mesenchymal interactions resulted in the formation of structures expressing markers of the outer and inner root sheaths [48].

A recent study obtained HF equivalents in vitro using synthetic guides, primary human keratinocytes, and dermal papilla cells obtained during hiPSC differentiation. Modified dermal papilla cells were placed into the guide simulating the hair sheath, followed by keratinocytes. After that, a nylon fiber was integrated into the guide. As a result of epithelial-mesenchymal interactions under conditions of proper spatial orientation and specified mechanical interactions between the system elements, an axial structure of the HF was formed, including analogs of the inner and outer root sheath [36].

The above models attempt to reproduce folliculogenesis using postnatal cells resulting from stimulation of epithelial-mesenchymal interactions using external mechanical guides and specific cytokines. These models are of interest in terms of studying transdifferentiation and migration of epithelial cells. However, all the models described above failed to complete full-sized hair shaft generation.

### 10.4. LSEs With Skin Derivatives From hiPSC Embryoid Bodies

Using a fundamentally different approach, it was possible to achieve generation of an HF and skin analog in vitro through the differentiation of embryoid bodies (spheroids from pluripotent stem cells) from mouse pluripotent stem cells (mPSCs). To ensure differentiation, a BMP4 and TGF-*β* signaling pathway inhibitor (SB431542) was introduced into the medium, resulting in the differentiation of the peripheral layer of the embryoid cell into surface ectoderm and that of the inner core layer into undifferentiated ectoderm. Under the action of FGF2 and LDN193189, a signaling pathway inhibitor of TGF-*β* and SMAD, a cell subpopulation similar to neural crest cells formed in the center, which soon differentiated into a number of mesenchymal and neuroglial subpopulations. During further development, the internal cell mass of the organoid migrated through the ectoderm to the outside, forming a cyst lined by epithelium inside the organoid. After that, the mesenchyme differentiated and formed an analog of the dermis, and the epithelial lining of the cyst formed an analog of the epidermis. As a result, epithelial-mesenchymal interactions within the organoid produced a skin fragment with HF-bearing shafts oriented into the interior of the formed structure (Figure 3) [34].

The next significant step in this direction was the successful application of the approach tested on mPSCs to hESCs and hiPSCs, using a similar set of factors (LDN193189, BMP4, FGF, and SB4312) to trigger differentiation in the right direction, but the timing and concentrations differed from the protocol for mPSCs. Under the influence of the above-mentioned factors, in the 2nd week of differentiation, the integumentary ectoderm and neuroectoderm were formed. During further differentiation, by the 15th day of development, a cavity lined with epithelial cells was formed in the center of the organoids. The mesenchyme and neural sockets formed by neural stem progenitor cells were formed within the organoids. During the first week, various glial subpopulations, including anterior and posterior placode cells, arose. By Day 100 of differentiation, the basal and spinous layers were formed in the epithelium. HFs and sebaceous glands also formed. Hair shafts of HFs were oriented into the interior of the dermal organoids, as in the case of the mPSC differentiation protocol. The organoids reached 2–3 mm in diameter. This was the first documented case of generation of complete human HFs capable of forming a hair shaft in vitro and the first described case of skin organoid generation with such a diverse set of other derivatives and cell subpopulations [35]. The resulting organoids were spheroids, consisting mainly of the dermis with a cavity lined by epithelium. Part of the organoid did not contain the dermis with cavity and consisted of cartilaginous tissue. Merkel cells, adipocytes, fibroblasts, melanocytes, and various glial cells were also found. The HFs included a dermal papilla and an axial structure similar to immature human HFs containing hair shaft keratins and layers of epithelial cells. In addition, the formed HFs were surrounded by a layer of fibroblasts, the dermal sheath. Smooth muscle cells, which are involved in the change of hair angle (*arrector pili*) in mammals, were found next to the HFs. After the transplantation of the obtained dermal organoids into the skin of immunodeficient mice, the dermis and epidermis of the dermal organoid were successfully incorporated into the dermis and epidermis of the skin of the host, the HFs being reoriented orthogonally to the skin surface during integration. No visible tumors, scars, or keloids were detected [35, 48].

Subsequently, using a modification of the protocol described above, skin organoids with a keratinized epidermis were obtained [160]. With a further modification, induction of Wnt signaling by introducing CHIR 99021, an agonist of the Wnt signaling pathway, on Day 4 of differentiation, it was possible to derive skin organoids without cartilage inclusions. By integrating the obtained dermal organoids into the classical two-component LSCEs and further culturing the resulting system in a hybrid air-liquid interface based on transwells, this study obtained a complete keratinized skin equivalent, including a dermal analog with integrated derivatives (HF, sebaceous glands, and smooth muscles) [48]. This LSE was the closest to its native analog at the time of this review. In 2023, another research team optimized the described protocol of embryoid body differentiation with the resulting generation of enhanced hair-bearing skin organoids with sebaceous glands [161].

Thus, the approach to LSE generation using directed differentiation of embryoid bodies from pluripotent cells in the ectodermal direction has reproducibly developed a structure similar to native skin. This model mimics the differentiation of ectoderm into the skin of the facial part of the human skull, since the skin in this area is completely formed from this germ layer [162]. The positive aspects of the method described above include the fact that the experiment was implemented without the use of serum or its substitute, or of expensive microfluidic systems [31]. Another advantage is the potential for successful cryopreservation due to the small size of the organoid (up to a few millimeters in diameter), which should facilitate the penetration of the cryoprotectant into the tissue [163]. Limitations of the method include the long time for three-dimensional differentiation (3–4 months before skin formation with HFs), dependence on both the genetic and epigenetic states of pluripotent cells, and on the quality of Matrigel®, whose composition and physical properties may change from batch to batch, and the use of expensive growth factors [31]. Further development of similar methods combining the use of dermal organoids and various biopolymer scaffolds may, in theory, make it feasible to obtain nearly native full-thickness LSEs [164]. The models described above have great potential for embryological studies of skin morphogenesis of ectodermal origin and for pharmacological research and alopecia therapy development.

## 11. LSCEs From Genetically Modified Cells

**Figure 4:**
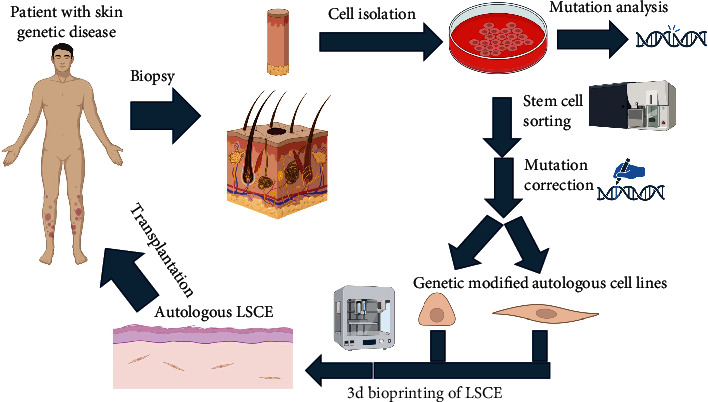
Strategy for application of genetic treatment of skin disease in medical practice. The figures were made using the online editor BioRender.

Another important direction in the development of the technology of using LSCEs for medical purposes is development and application of LSCEs from genetically modified cells, in which a defect associated with inherited or acquired mutations or combinations of certain allelic genes has been eliminated. Examples of relevant diseases are epidermolysis bullosa, melanoma, ichthyosis, and xeroderma pigmentosum [165]. Despite the variety of genetic modification methods tested on cell cultures and aimed at correcting genetic defects leading to the above diseases, few examples of LSCEs are based on genetically modified cell lines, and there are very few instances of using these constructs clinically (Figure 4).

One of the most pressing areas within this topic is the generation of genetically modified LSCEs created for the replacement therapy of epidermolysis bullosa [166], a dangerous genetic skin disease in which the formation of the normal basal lamina proteins is disrupted. Work on editing the keratinocytes of patients with epidermolysis bullosa began in the 1990s, aiming to form a stable cell line expressing a copy of the corrected COL17A1, LAMB3, ITGB4, K5, and K14 genes [167–170]. LSCEs based on autologous genetically modified keratinocytes have been produced and tested for clinical purposes. A 7-year-old boy suffering from epidermolysis bullosa underwent a series of autologous skin equivalent transplants consisting mainly of posttransduction keratinocytes (the retroviral vector with LAMB3 cDNA), transplanting almost the entire skin surface of the child (0.85 m^2^). No visible tumors, scars, or keloids were detected. Laminin expression was maintained in the genetically corrected keratinocytes for 3 years, and no pathological skin blisters were detected on the patient's body surface [171]. Work in this direction continues.

## 12. Discussion

As a result of half a century of continuous development in cell engineering, stem cell biology, molecular biology, and embryology, technologies have been developed to produce both single cell lines phenotypically resembling subpopulations of cells within the skin and its derivatives, such as epidermal stem cells, fibroblasts, DP cells, and melanocytes, and LSEs that maximally reproduce the dermis and epidermis structure of the skin, including skin derivatives such as HF. New biomaterials, technologies, and approaches have made it possible to create simple and complex LSCEs for a range of applications. New additive methods for the construction of LSEs, such as bioprinting, electrospinning, and new approaches to the cultivation of LSEs, including microfluidic systems on chips and bioreactors, show improvements in time and quality of equivalent generation. These have the potential to minimize manual manipulation and to reduce the price of skin graft construction. 3D and 4D bioprinting can potentially be used for rapid generation of massive LSCEs to cover large wound areas. Electrospinning allows the generation of unique new matrices and substrates for LSCEs. New approaches such as the combination of gene therapy and LSEs with genetically modified cell generation and transplantation are able to solve some problems in modern translational medicine and can be used for the treatment of some genetic skin diseases, for example, epidermolysis bullosa. Microfluidic systems are aimed at long-term cultivation of massive, complex, and full-thickness LSCEs. However, all these technologies are currently too bulky for medical and mass production applications and require further steps for safety approval and laboratory optimization.

Current LSE models based on embryoid cell differentiation show great efficiency in generating complex LSEs that maximally resemble native human skin in their properties. However, they have the following major limitations: problems with gas exchange, nutrient access, excretion of metabolic products and self-maintenance during prolonged cultivation, and high cost. Thus, as current classical LSE models are suitable for limited medical applications and are used in commercial solutions, they remain relevant. Most modern LSEs are still unable to reconstruct all the derivatives and cell types that may be present in the human skin. To address these problems, further development of 3D and 4D bioprinting technologies can be expected, based on existing technologies for generating LSEs from embryoid bodies using computer modeling, which will allow customization of shape, size, and other properties. Other protocols for differentiation of embryoid bodies can be expected, enabling the development of skin organoids with different properties, structures, and sets of derivatives. The development of advanced technologies in this field of translational medicine, as well as the generation of new approaches, will make it possible to achieve these goals and fulfill the requirements for an ideal LSE (Figure 2). The skin and blood have been among the first objects of cell technologies and tissue engineering. Several technologies aimed at obtaining LSEs can also be used to create equivalents of other organs whose composition includes epithelium and connective tissue.

## 13. Conclusion

In this review, both classical and modern technologies for the production of LSCEs and new types of LSCEs have been described. However, the transfer of the latest developments into practical medicine is currently not very active. This is hampered by the problems of protocols that are difficult to reproduce using expensive reagents and rare equipment. Innovative technologies and approaches can be used to obtain multicomponent LSCEs that are close to their native analogs and hold great promise for pharmacological studies, process modeling, and scientific research. In the future, it may be possible to use them to create ideal LSCEs for regenerative medicine.

Classical LSEs, produced according to well-established protocols using well-studied matrices such as collagen gel or hyaluronic acid in combination with allogeneic cellular material, are still relevant and have potential for development.

## Figures and Tables

**Table 1:** Examples of commercial LSCEs for clinical use and spheres of their application.

**Type of LSCE**	**Commercial product and manufacturer**	**Cell component**	**Matrix**	**Sphere of application**
Epidermal LSCEs	Epicel® Vericel, Cambridge, USA	Autologous epidermal keratinocytes	—	Adjuvant in burn treatment

Dermal LSCEs	TransCyte® Shire Regenerative Medicine, Inc., San Diego, CA, USA; Smith & Nephew, Inc., Largo, FL, USA	Human allogeneic fibroblasts derived from the foreskin of a newborn	Silicone-coated biodegradable nylon sponge	Temporary coverage for burns, chronic and diabetic ulcer treatment
	Dermagraft® Shire Regenerative Medicine, Inc., San Diego, USA	Frozen allogeneic neonatal fibroblasts, obtained from newborns	Bioresorbable collagen on a sponge from polyglactin (Dexon) or Polyglactin 910 (Vicryl)	Temporary coverage for deep diabetic ulcers, chronic, and noninfectious wound treatment

Full-thickness LSEs	Apligraf®/Graftskin® Organogenesis, Canton, USA	Epidermal keratinocytes from the foreskin of newborns and human neonatal fibroblasts	Gel from bovine Collagen I with fibroblasts	Temporary coverage for chronic congestive venous ulcers and deep ulcers, epidermolysis bullosa treatment, recovery after repeated hernias, bedsores, reconstruction of burn injuries
	OrCel® Forticell Bioscience, New York City, USA	Epidermal keratinocytes and fibroblasts from the foreskin of newborns and human neonatal	Layers of gel from bovine Collagen I with epidermal cells and fibroblasts	Temporary coverage for dystrophic epidermolysis bullosa symptom treatment, autotransplantation to donor sites in burn patients
	Laserskin® Fidia Advanced Biopolymers Abano Terme, Italy	Autologous keratinocytes and fibroblasts from skin biopsy	Biodegradable matrix from esterified hyaluronic acid	Temporary coverage for diabetic ulcers and deep skin wound treatment, drainage of fluid from a wound
	PermaDerm® Regenicin, Inc., Little Falls, USA	Autologous keratinocytes and fibroblasts, cultured on bovine collagen scaffold	—	Permanent coverage of burn areas as skin replacement

	StrataGraft® The Luminis Group, Ltd. for Stratatech Corp., Madison, USA	Immortalized keratinocytes NIKS® and dermal line fibroblasts on a collagen scaffold	—	Permanent coverage of burn and chronic ulcer areas as skin replacement

**Table 2:** Examples of commercial LSCEs for research and pharmacology tests.

**Type of LSCE**	**Commercial product and manufacturer**	**Cell component**	**Matrix**	**Sphere of application**
Epidermal LSCEs	SkinEthic Rhe®, Episkin, Lyon, France	Human epidermal keratinocytes (stratification in air-liquid interface)	—	Tests: - Pharmacological tests of drugs - DNA damage - UV exposure - Bacterial adhesion - Permeability - Cryopreservation protocols optimization - Wound healing - Skin hydration and dehydration
	Epiderm®, MatTek, Ashland, USA	Human epidermal neonatal keratinocytes (stratification in air-liquid interface)	—	
	Epidermal skin test 1000 (EST1000), CellSystems Biotechnologie GmbH, Troisdorf, Germany	Human primary epidermal keratinocytes (stratification in air-liquid interface)	—	
Full-thickness LSEs	SkinEthic®, Episkin, Lyon, France	Human epidermal keratinocytes (stratification in air-liquid interface)	Collagen I gel	
	LabSkin® University of Bradford's Centre for Skin Sciences, Bradford, UK	Human epidermal keratinocytes (stratification in air-liquid interface) and fibroblasts	Fibrin matrix with fibroblast	
	EpidermFT®, MatTek, Ashland, USA	Human epidermal neonatal keratinocytes (stratification in air-liquid interface) and fibroblasts	Collagen I gel with fibroblasts	
	StrataTest®, StrataTech, Madison, USA	Human epidermal near diploid keratinocytes, NIKS® (stratification in air-liquid interface) and fibroblasts	Collagen I gel with fibroblasts	
	Advanced skin test 2000 (EST2000), CellSystems Biotechnologie GmbH, Troisdorf, Germany	Human epidermal primary keratinocytes (stratification in air-liquid interface) and fibroblasts	Collagen I gel with fibroblasts	

## Data Availability

This research did not generate or analyze any data.
